# Autoantibodies Profile in the Sera of Patients with Sjogren]s Syndrome: The ANA Evaluation—A Homogeneous, Multiplexed System

**DOI:** 10.1080/10446670410001670490

**Published:** 2004-03

**Authors:** Boris Gilburd, Mahmoud Abu-Shakra, Yehuda Shoenfeld, Andrea Giordano, Elena Bartoloni Bocci, Francesco delle Monache, Roberto Gerli

**Affiliations:** 1 Department of Medicine B Center for Autoimmune Diseases Sheba Medical Center Sackler Faculty of Medicine, Tel-Aviv University. Tel-hashomer Israel; 2 Autoimmune Rheumatic Disease Unit Soroka Medical Center and Ben-Gurion University Beer-Sheva Israel; 3 Section of Internal Medicine and Oncological Services Department of Clinical and Experimental Medicine Center for Study of Rheumatic Diseases University of Perugia Perugia Italy

## Abstract

*Background*: Flow-based, multiplex bead arrays (MBA) have been developed for a variety of applications including the detection of antibodies to extractable nuclear antigens (ENA). It offers a rapid and sensitive method to assess multiple analyses in a single tube/well.

*Purpose*: To evaluate the Athena Multi-Lyte ANA Test System utilizes Luminex Corporation's MBA technology for the detection of antinuclear antibodies (ANA) and ENA antibodies in the sera of patients with Sjogren's syndrome (SS).

*Methods*: MBA assay was used to detect ANA and ENA antibodies in the sera of 37 patients with SS and 96 sera from healthy subjects.

*Results*: All patients were women. Their mean age was 48.7 years and the mean disease duration was 7.27 years. ANA was found in 3 (3%) sera of healthy subjects by the AtheNA system and in 2 (2%) sera by the ELISA kit. A 99% concordance between the 2 assays was found. A 94.6% concordance between the 2 assays was found by testing the sera of patients with SS for ANA.

By the AtheNA system, none of the sera of 37 patients with SS had autoantibodies reacting with Sm, Jo-1, dsDNA or histones. Anti-RNP antibody was found in 5.4% of the sera and 2.7% of the sera reacted with Scl-70 and histones. Anti-SS/A and anti-SS/B were identified in 84 and 76% of the sera, respectively.

*Conclusion*: The AtheNa Multi-Lyte ANA Test System offers a sensitive and specific result for the detection of ANA and ENA antibodies in the sera of patients with SS.


*Clinical & Developmental Immunology*, March 2004, Vol. 11 (1), pp. 53–56

Taylor & Francis healthsciences

### Autoantibodies Profile in the Sera of Patients with Sjogren's Syndrome: The ANA Evaluation—A Homogeneous, Multiplexed System

BORIS GILBURD[a], MAHMOUD ABU-SHAKRA[b], YEHUDA SHOENFELD[a,*], ANDREA GIORDANO[c], ELENA BARTOLONI BOCCI[c], FRANCESCO DELLE MONACHE[c] and ROBERTO GERLI[c]

[a]*Department of Medicine B, Center for Autoimmune Diseases, Sheba Medical Center, Sackler Faculty of Medicine, Tel-Aviv University, Tel-Hashomer, Israel;* [b]*Autoimmune Rheumatic Disease Unit, Soroka Medical Center and Ben-Gurion University, Beer-Sheva, Israel;* [c]*Section of Internal Medicine and Oncological Services, Department of Clinical and Experimental Medicine, Center for Study of Rheumatic Diseases, University of Perugia, Perugia, Italy*

*Background*: Flow-based, multiplex bead arrays (MBA) have been developed for a variety of applications including the detection of antibodies to extractable nuclear antigens (ENA). It offers a rapid and sensitive method to assess multiple analyses in a single tube/well.

*Purpose*: To evaluate the Athena Multi-Lyte ANA Test System utilizes Luminex Corporation's MBA technology for the detection of antinuclear antibodies (ANA) and ENA antibodies in the sera of patients with Sjogren's syndrome (SS).

*Methods*: MBA assay was used to detect ANA and ENA antibodies in the sera of 37 patients with SS and 96 sera from healthy subjects.

*Results*: All patients were women. Their mean age was 48.7 years and the mean disease duration was 7.27 years. ANA was found in 3 (3%) sera of healthy subjects by the AtheNA system and in 2 (2%) sera by the ELISA kit. A 99% concordance between the 2 assays was found. A 94.6% concordance between the 2 assays was found by testing the sera of patients with SS for ANA.

By the AtheNA system, none of the sera of 37 patients with SS had autoantibodies reacting with Sm, Jo-1, dsDNA or histones. Anti-RNP antibody was found in 5.4% of the sera and 2.7% of the sera reacted with Scl-70 and histones. Anti-SS/A and anti-SS/B were identified in 84 and 76% of the sera, respectively.

*Conclusion*: The AtheNa Multi-Lyte ANA Test System offers a sensitive and specific result for the detection of ANA and ENA antibodies in the sera of patients with SS.

*Keywords*: Sjogren's syndrome; Autoantibodies; Multiplex bead arrays; Extractable nuclear antigens (ENA)

Sjogren's syndrome (SS) is an autoimmune disease with distinct clinical and laboratory features. Cardinal features of the disease include focal mononuclear cell infiltration of exocrine tissues, the production of a wide variety of autoantibodies, and oral and ocular symptoms and signs of dryness (Fox *et al.*, 2000). In addition, the patients with SS may present with respiratory, cutaneous, musculoskeletal, central nervous system, renal and other vital organs involvement. Laboratory features of SS include hyperglobulinemia, presence of: rheumatoid factor, antinuclear antibodies (ANA), anti-SS/A and -SS/B autoantibodies (Cervera *et al.*, 2000).

Recently, flow-based, multiplex bead arrays (MBA) have been developed for a variety of applications including the detection of extractable nuclear antigens (ENA) antibodies. This platform offers the potential for a rapid and sensitive method with reduced hands-on time required for performance because of the ability to assess multiple analytes in a single tube/well (Fulto *et al.*, 1997; Dario, 2000; Prabhakar *et al.*, 2002; Rouquette *et al.*, 2003). AtheNA multi-lyte test system is a homogeneous, multiplexed system for autoantibodies analysis. It is precise and easy to use technology for simultaneous quantitative multi-autoantibody detection in a single microtiter well.

The purpose of this study was to evaluate the Athena Multi-Lyte ANA Test; an MBA technology for the detection of ANA and ENA antibodies in the sera of patients with SS and healthy subjects. In addition to determine the concordance between AtheNA and standard ELISA assays in detecting of antinuclear antibodies.

*Corresponding author. E-mail: shoenfel@post.tau.ac.il

ISSN 1740-2522 print/ISSN 1740-2530 online © 2004 Taylor & Francis Ltd
DOI: 10.1080/10446670410001670490

### PATIENTS AND METHODS

### Patients

The study population comprised of 37 consecutive patients with SS. All patients fulfilled the criteria of classification of SS (Vitali *et al.*, 2002). Patients were seen at 3–4 months intervals.

### Autoantibody Determination

AtheNA Multi-Lyte ANA Test System (Zeus Scientific, Inc. Raritan, NJ 08869, USA) was used for simultaneous determination of autoantibodies to nine different analyts (SS/A, SS/B, Sm, RNP, Scl 70, Jo-1, dsDNA, Centromere B and Histone).

ANA in sera of patients and control subjects was evaluated by the AtheNA Multi-Lyte ANA Test System and by AESKULISA ANA-8S ELISA assay (Aesku.lab Diagnostika Mikroforum Ring 2 55234 Wendelsheim Germany).

Positive tests for all individual autoantibodies in the AtheNA Multi-Lyte ANA Test System were defined as values greater than 120 units/ml (cutoff normal range). For the ANA tests, values $>1.2$ were defined as positive.

### Controls

Sera of age matched apparently healthy women were obtained from national blood bank and were also tested by the AtheNA system for ANA and the various autoantibodies.

### Statistical Analyses

Statistical analysis was carried out using the STAT program. Descriptive statistics and linear correlation were used to correlate between the various assays.

### RESULTS

The demographics and main clinical features of the primary SS patients included in the study are shown in Table I. All patients were women. Their mean age at diagnosis of SS was 48.7 years (27–78) and their mean disease duration was 7.27 years (1–15) from diagnosis and 10.9 (range 3–23) from symptoms onset.

All patients had symptoms and objective signs of both ocular and oral sicca syndrome. Other main clinical features included are arthritis (28 patients, 76%), recurrent parotid enlargement (15 patients, 41%), Raynaud's phenomenon (5 patients, 14%) and serositis (3 patients, 8%). None of the patients had malignancy, neurological, cardiac or renal involvement.

Laboratory features included are leukopenia (17 patients, 46%), rheumatoid factor positivity (31 patients, 84%), hypergammaglobulinemia (21 patients, 57%) and monoclonal gammopathy in 3 patients (8%).

TABLE I Demographics and clinical features of the patients with Sjogren's syndrome

| | |
|---|---|
| Gender | |
| Female(%) | 100 |
| Age | |
| Years (range) | 48.7 (27–78) |
| *Disease duration years (range)* | |
| From symptoms onset | 10.95 (3–23) |
| From diagnosis | 7.27 (1–15) |
| Main clinical features | |
| Ocular | 37/37 |
| Oral | 37/37 |
| Recurrent parotid enlargement | 15/37 |
| Arthritis/arthralgia | 28/37 |
| Serositis | 3/37 |
| Vasculitic purpura | 2/37 |
| Raynaud's phenomenon | 5/37 |
| Autoimmune hepatitis | 2/37 |
| Autoimmune thyroiditis | 3/37 |
| Leucopenia ($<4.000/mm^3$) | 17/37 |
| Rheumatoid factor + | 31/37 |
| Polyclonal | 21/37 |
| MGUS* | 3/37 |
| Neurological involvement | 0/37 |
| *Drugs* | |
| Corticosteroids (5–7.5 mg for less than 3 months) | |
| Current | 0/37 |
| Previous | 13/37 |
| NSAIDs | |
| Current | 6/37 |
| Previous | 7/37 |
| Hydroxychloroquine | |
| Current | 13/37 |
| Previous | 2/37 |

*Monoclonal gammopathies of unknown significance.

Medical treatment given during the disease course are shown in Table I.

### Autoantibody Profile

#### *Normal Sera*

The sera of 98 apparently normal individuals, obtained from national blood bank were tested by the AtheNA system and by AESKU Diagnostics ANA-8S ELISA Kit. ANA was found in 3 (3%) sera from healthy subjects by the AtheNA system and in 2 (2%) sera by the ELISA kit. A 99% concordance between the 2 assays was found.

All sera were tested by the AtheNA for autoantibodies reacting with: SS/A, SS/B, Sm, RNP, Scl-70, Jo-1, dsDNA, Centromere and histones. None of the sera had anti-SS/A, anti-SS/B, anti-Jo-1, anti-dsDNA, anti-centromere or anti-histone activity.

Anti-Sm, anti-RNP and anti-Scl-70 were detected each in one (1%) serum.

#### *Sera of Patients with SS*

Table II shows the percentage of patients with SS and healthy controls who had ANA by the AtheNA system and by the AESKU Diagnostics ANA-8S ELISA kit. A 94.7% concordance between the 2 assays was found by testing the sera of patients with SS.

TABLE II Evaluation of ANA screening by AtheNA multiplex system and standard ELISA kit

| Group | AtheNA No (%) | ELISA No (%) | Concordance (%) |
|---|---|---|---|
| *Healthy controls* | | | |
| Positive | 93 | 94 | 99 |
| Negative | 3 | 2 | |
| *Patients with Sjorgen's syndrome* | | | |
| Positive | 32 | 30 | |
| Negative | 5 | 7 | 94.6 |

By the AtheNA system, none of the sera of 37 patients with SS had autoantibodies reacting with Sm, Jo-1, dsDNA or histones. Anti-RNP antibody was found in 5.4% of the sera (2 patients) and 2.7% of the sera (1 patient) reacted with Scl-70 and histones.

Anti-SS/A and -SS/B, which are highly associated with SS, were identified in 84% (31 patients) and 76% of the sera (28 patients), respectively. Their titers are shown in Fig. 1.

### DISCUSSION

During the last 3 decades a wide range of autoantibodies has been identified in the sera of patients with various autoimmune diseases. In a recent review, (Sherer *et al.*) more than 100 autoantibodies reported in the sera of patients with systemic lupus erythematosus (SLE). A significant number of those autoantibodies were found to be pathogenic and has a diagnostic and/or activity role in various autoimmune diseases (Cervera and Shoenfeld, 1996). In a substantial number of cases the diagnosis of autoimmune disease is confirmed by the detection of specific autoantibodies. Therefore, rapid and sensitive methods are needed to allow prompt identification of autoantibodies and early diagnosis of autoimmune diseases.

Recently, flow-based, MBA have been developed for detection of a variety of autoantibodies including ANA, anti-sdDNA, anti-ENA, anti-histones, antibodies reacting with various ribo-nuclear proteins and other autoantibodies. This system allows evaluating multiple autoantibodies in a single tube or well (Rouquette *et al.*, 2003). In a recent study, the sera of patients with various autoimmune rheumatic diseases were tested for 15 autoantibodies in microarrays. The data of the microarray system were similar to those by conventional method (Feng *et al.*, 2003).

The AtheNA multi-lyte Test System was developed by Zeus Scientific (Raritan, NJ). It is a homogeneous, multiplexed system for autoantibodies analysis. It is highly precise and easy to use technology for simultaneous quantitative multi-autoantibody detection in a single microtiter well.

In the present study, we found a 99 and 94.7% concordance between AtheNA and standard ELISA assay for detection of ANA in the sera of apparently healthy individuals and sera of patients with SS, respectively. The data suggest that the AtheNA Multi-Lyte ANA Test System offers a sensitive and specific instrument for the detection of ANA in the sera of healthy individuals and patients with an autoimmune disease.

In addition, the sera of the patients with SS and healthy controls were also tested by AtheNA for a various autoantigens including dsDNA, histones, RNP, Sm, SS/A, SS/B, Jo-1 and centromere.

In the sera of the normal individuals, no autoantibody activity was detected in the vast majority of sera. Only 3 individuals had autoantibodies in their sera. One individual had anti-Sm, other one had anti-RNP and a third person had anti-Scl-70, all of them were in low-medium titers.

The presence of autoantibodies in the sera of normal individuals in low-medium titers has been reported in numerous reports. Usually, those autoantibodies are poly-specific, belong to the IgM isotype, and bind their autoantigens with low affinity and avidity (Abu-Shakra and Shoenfeld, 1993). All of these features are characteristics of natural autoantibodies. The frequency of autoantibodies in normal individuals ranges between 0.5 and 27% (Abu-Shakra and Shoenfeld, 1993).

The sera of the patients with SS reacted mainly with SS/A and SS/B. Eighty-four and 74% of the sera of the SS patients had medium-high titer of anti-SS/A and -SS/B antibodies. A literature review has found the reported frequency of anti-SS/A and anti-SS/B in the sera of patients with SS to be between 50–90% (Bell *et al.*, 1999; Cervera and Shoenfeld, 1996).

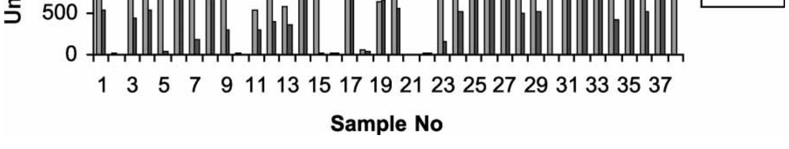

FIGURE 1 Anti SS/A and anti-SS/B in the sera of patients with SS.

Taken together, the data suggest that the sensitivity and the highly precise technology of the AtheNA evaluation, makes it feasible and easy to conduct the simultaneous detection of quantitive multiple autoantibodies in a single micro titer, in the sera of patients with SS and in the sera of normal individuals.

### References

Abu-Shakra, M. and Shoenfeld, Y. (1993) "Introduction to natural autoantibodies", In: Shoenfeld, Y. and Isenberg, D., eds, Natural Autoantibodies (CRC Press), p 1533.

Bell, M., Askari, A., Bookman, A., *et al.* (1999) "Sjogren syndrome: a critical review of clinical management", *J. Rheumatol.* **26**, 2051–2061.

Cervera, R. and Shoenfeld, Y. (1996) "Pathogenic mechanisms", In: Peter, J. and Shoenfeld, Y., eds, Autoantibodies (Elsevier), pp 607–617.

Cervera, R., Font, J., Ramos-Casals, M., Garcia-Carrasco, M., Rosas, J., Morla, R.M., Munoz, F.J., Artigues, A., Pallares, L. and Ingelmo, M. (2000) "Primary Sjogren's syndrome in men: clinical and immunological characteristics", *Lupus*, 61–64.

Dario, A.A. and Vignali (2000) "Multiplexed particle-based flow cytometric assays", *J. Immunol. Meth.* **243**, 243–255.

Feng, Y., Ke, X., Ma, R., Chen, Y., Hu, G. and Liu, F. (2004) "Parallel detection of autoantibodies with microarrays in rheumatic diseases", *Clin. Chem.* **50**, 416–422.

Fox, R.I., Stern, M. and Michelson, P. (2000) "Update in Sjogren syndrome", *Curr. Opin. Rheumatol.* **12**, 391–398.

Fulto, R.J., McDade, R.L., Smith, P.L., Kienker, L.J. and Kettman, J.R. (1997) "Advanced multiplexed analysis with the FlowMetrixTM system", *Clin. Chem.* **43**, 1749–1756.

Prabhakar, U., Eirikis, E. and Davis, H.M. (2002) "Simultaneous quantification of proinflammatory cytokines in human plasma using the LabMAP2 assay", *J. Immunol. Meth.* **260**, 207–218.

Rouquette, A.M., Desgruelles, C. and Laroche, P. (2003) "Evaluation of the new multiplexed immunoassay, FIDIS, for simultaneous quantitative determination of antinuclear, antibodies and comparison with conventional methods", *Am. J. Clin. Pathol.* **120**, 676–681.

Sherer, Y., Gorstein, A., Fritzler, M., Shoenfeld, Y., "Autoantibody explosion in SLE: More than 100 different antibodies found in SLE patients", *Semin. Arthritis Rheum.*, In press.

Vitali, C., Bombardieri, S., Jonsson, R., *et al.* (2002) "Classification criteria for Sjogren's syndrome: a revised version of the European criteria proposed by the American-European Consensus Group", *Ann. Rheum. Dis.* **61**, 554–558.

